# When to Eat Meat

**Published:** 1883-01

**Authors:** 


### WHEN TO EAT MEAT.

It is well known that the digestion of different kinds of meat is the more easily effected the longer the time that transpires after the death of the animal. The explanation given is, that by keeping, the muscular fibres become gradually dissociated ; they soften, become less compact, and consequently are more soluble in the gastric juice. According to physiologists, however, it is not considered advisable to wait until decomposition sets in, because, in addition to its losing a great part of its nutritive qualities, the meat becomes so nauseous that no amount of cooking, or the addition of condiments, will much improve it. MM. Pasteur and Lemaire, in an interesting paper lately submitted by them to the Academy of Sciences, stated that meat too far advanced, or what is termed "faisandée," is most unwholesome, and it is a mistake committed daily by sportsmen to wait until the game gets into this condition, for it is then simply unfit to be eaten. The above-named biologists have shown that tainted meat contains animalcules, which do the work of transformation and destruction ; and as it is difficult to ascertain exactly the extent of putrefaction that the meat has undergone, one is liable to consume it just at the moment when it should be rejected. M. Pasteur and other micrographers are of the opinion that these animalcules, of which there are no less than thirty species, are of the same nature as those that are found in living animals suffering from virulent maladies, such as charbon, etc.—*Lancet.*

